# Supplementary material for: The interactome of intact mitochondria by cross-linking mass spectrometry provides evidence for coexisting respiratory supercomplexes
Source: Mol Cell Proteomics. 2017 Dec 8;17(2):216–32. doi: 10.1074/mcp.RA117.000470 (PMC5795388; doi:10.1074/mcp.RA117.000470)
Supplement: Supplemental Data [file supp_RA117.000470_133922_0_supp_23978_fzffwf.zip › spectra_annotation/mito_spectra_annotation/77-1-5-1-7-1.pdf]

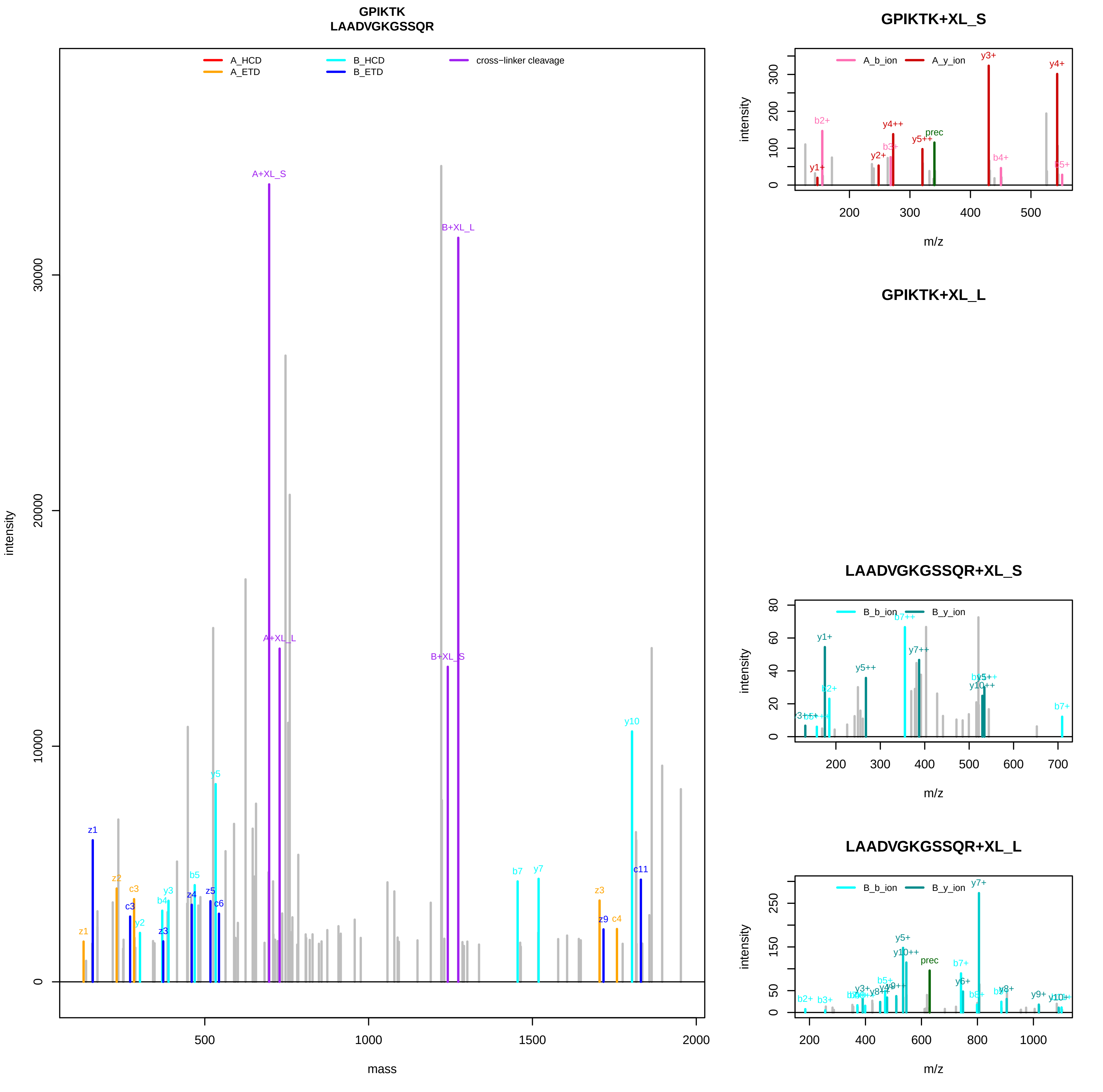
GPIKTK
LAADVGKGSSQR
A_HCD
A_ETD
B_HCD
B_ETD
cross−linker cleavage
A+XL_S
B+XL_L
A+XL_L
B+XL_S
intensity
mass
0
10000
20000
30000
500
1000
1500
2000
z1
z2
c3
z1
y2
y3
b4
z3
z4
b5
z5
y5
c6
b7
y7
z3
z9
c4
c11
y10
GPIKTK+XL_S
A_b_ion
A_y_ion
y1+
b2+
y2+
y4++
b3+
y5++
prec
y3+
b4+
y4+
b5+
m/z
GPIKTK+XL_L
LAADVGKGSSQR+XL_S
B_b_ion
B_y_ion
y1+
b2+
y5++
b7++
y7++
y10++
b7+
LAADVGKGSSQR+XL_L
y7+
y5+
y10++
prec
b7+
y6+
b5+
y3+
y8+
y9+
